# Occurrence of some common carbapenemase genes in carbapenem-resistant *Klebsiella pneumoniae* isolates collected from clinical samples in Tabriz, northwestern Iran

**DOI:** 10.1186/s13104-023-06558-x

**Published:** 2023-11-03

**Authors:** Abolfazl Jafari-Sales, Noor S.K. Al-Khafaji, Hussein O.M. Al-Dahmoshi, Zahra Sadeghi Deylamdeh, Sousan Akrami, Afsoon Shariat, Hawraa K. Judi, Rozita Nasiri, Hossein Bannazadeh Baghi, Morteza Saki

**Affiliations:** 1 Department of Microbiology, School of Basic Sciences, Kazerun Branch, Islamic Azad University, Kazerun, Iran; 2 Department of Biology, College of Science, University of Babylon, Babylon, Hilla City, Iraq; 3 Department of Biology, Faculty of Sciences, Malayer Branch, Islamic Azad University, Malayer, Iran; 4 Students’ Scientific Research Center (SSRC), Tehran University of Medical Sciences, Tehran, Iran; 5 Department of Microbiology, Faculty of Medicine, Ahvaz Jundishapur University of Medical Sciences, Ahvaz, Iran; 6 Department of Medical Physics, Hilla University College, Babylon, Iraq; 7 Iran National Elite Foundation, 93111-14578 Tehran, Iran; 8 Infectious and Tropical Diseases Research Center, Tabriz University of Medical Sciences, Tabriz, Iran

**Keywords:** Carbapenem-resistant, Carbapenemase, Drug resistance, *Klebsiella pneumoniae*

## Abstract

**Objectives:**

This study aimed to evaluate the antibiotic resistance patterns and prevalence of carbapenemase genes in *Klebsiella pneumoniae* isolates in different clinical samples from Tabriz city, northwestern Iran.

**Results:**

This cross-sectional study was conducted in the Department of Microbiology, Islamic Azad University, Ahar Branch, Iran, in 2020. *K. pneumoniae* isolates were collected from different clinical samples, including blood, wounds, sputum, and urine. The isolates were identified using a series of standard bacteriological tests. Antibiotic resistance was determined by the disc diffusion method. The presence of *bla*_VIM_, *bla*_NDM_, *bla*_KPC_, *bla*_OXA_, and *bla*_IMP_ genes were screened by polymerase chain reaction (PCR). A total of 100 non-duplicated *K. pneumoniae* isolates were collected from 57 urine samples, 27 blood samples, 13 wound samples, and 3 sputum samples. Overall, 70.0% of the samples were from inpatients, while 30.0% were from outpatients. The most resistance rate was related to ampicillin (94.0%), while the lowest resistance rate was related to imipenem (18.0%) and meropenem (20.0%). Overall, 25.0% of the isolates were carbapenem-resistant, of which 13.0% were resistant to both imipenem and meropenem. The PCR showed the total prevalence of 23.0% for carbapenemase genes, including 18.0% for *bla*_KPC_, 3.0% for *bla*_VIM_, 1.0% for *bla*_IMP_, and 1.0% for *bla*_OXA_ gene. The *bla*_NDM_ gene was not detected in any isolate. The prevalence of carbapenemase-producing *K. pneumoniae* isolates was relatively lower in northwestern Iran than in other regions of the country. However, special attention should be paid to the proper use of antibiotics, particularly carbapenems, to prevent further spread of antibiotic resistance and its related genes.

## Introduction

*Klebsiella pneumoniae* is an opportunistic bacterium that can cause various infections [1, 2]. Carbapenem-resistant *K. pneumoniae* (CR-Kp) isolates are considered “critical concern” by the World Health Organization (WHO) [1, 2]. Carbapenemases are beta-lactamases capable of hydrolyzing the oxy-amine side chains of carbapenem antibiotics. *K. pneumoniae* carbapenemase (KPC), oxacillinase (OXA), Verona integron-encoded metallo-β-lactamase (VIM), imipenamas (IMP), and New Delhi metallo-β-lactamase (NDM) are among the most common carbapenemase genes in *K. pneumonia* [3, 4].

Since there are scare data on the prevalence of carbapenemase genes in CR-Kp from the northwestern region of Iran, this study aimed to evaluate the antibiotic resistance patterns and prevalence of carbapenemase genes in *K. pneumoniae* isolates from different clinical samples in Tabriz city, northwestern Iran. The results of this study may provide a better background for local antimicrobial prescribing protocols, local empirical treatments, and infection control programs.

## Main text

## Materials and methods

### Ethics approval and consent to participate

This study was approved by the IRB of the Kazerun Branch of the Islamic Azad University, Kazerun, Iran in accordance with the World Medical Association Declaration of Helsinki (no registered code). Clinical samples were collected as the routine laboratory analysis and to check any potential infection for referred and admitted patients and not as a part of this study. Therefore, written informed consent was waived by the IRB of the Kazerun Branch of the Islamic Azad University, Kazerun, Iran.

### Clinical isolates collection

This cross-sectional study was performed on 100 *K. pneumoniae* isolates over a six-month period (from June to November 2020). The isolates were collected from different clinical samples of inpatients and outpatients referred to hospitals in Tabriz, northwestern Iran. None of the patients had taken antibiotics three days prior to sample collection. Also, none of the patients had any underlying disease. Clinical samples included blood, wounds, sputum, and urine. The 100 *K. pneumoniae* strains were isolated from 49 female and 51 male patients, respectively. The mean age of the patients was 47.4 ± 23 years and ranged from a minimum of 10 months to a maximum of 70 years.

### Identification of *K. pneumoniae* isolates

Clinical samples were cultured on blood agar and MacConkey agar (Quelab, Canada) plates. The plates were incubated at 37 °C for 24 h. The grown colonies were confirmed by standard bacteriological tests, which included Gram stain, lysine iron agar (LIA), triple sugar iron agar (TSI), SIM (sulfide-indole- motility), Simon citrate, MR-VP (Methyl Red-Voges Proskauer), and urea broth [5]. The confirmed *K. pneumoniae* isolates were suspended in trypticase soy broth (TSB, Merck, Germany) with 20% (v/v) glycerol and stored at -80 °C for further investigations. *K. pneumoniae* ATCC® 13,883™ was used as quality control strain. All media were purchased from Merck Co, Germany.

### Antimicrobial susceptibility testing (AST)

The disc diffusion method was performed on Mueller-Hinton (MH) agar (Merck, Germany) for antibiotic susceptibility testing of *K. pneumoniae* based on the Clinical and Laboratory Standards Institute (CLSI) guidelines [6]. Antibiotics used included cephalothin (30 µg), imipenem (10 µg), meropenem (10 µg), ceftazidime (30 µg), ciprofloxacin (5 µg), cefoxitin (30 µg), gentamicin (10 µg), amikacin (30 µg), nalidixic acid (30 µg), ampicillin (10 µg), cotrimoxazole (23.75 µg), cefotaxime (30 µg), ceftriaxone (30 µg), tetracycline (30 µg), and azteronam (30 µg) (Himedia Co. India). Isolates that showed resistance to 3 or more antibiotic categories were classified as multidrug resistant (MDR) [7]. *Escherichia coli* ATCC® 25,922™ and *K. pneumoniae* ATCC® 13,883™ were used as quality control strains.

### Identification of carbapenemase genes

DNA was extracted from carbapenem-resistant isolates using the Invitek Strateg Business kit (Invitek-Molecular, Germany) according to the manufacturer instruction. Then uniplex conventional polymerase chain reaction (PCR) was performed using previously described specific oligonucleotide primers of *bla*_VIM_, *bla*_NDM_, *bla*_KPC_, *bla*_OXA,_ and *bla*_IMP_ genes (Table 1) [8, 9]. The final reaction mixture was 25 µl including 12.5 µl of Buffer 10X PCR, 1 mg/µl of MgCl_2_, 0.75 µM of deoxynucleotide triphosphates (dNTPs) mix, 1 µl of each primer, 0.2 units of Taq Polymerase, 3 µl of DNA, and the DNA/RNA free water. The PCR program was performed in a thermocycler (Eppendorf Master Cylinder, Germany) as follows: initial denaturation for 10 min at 94 °C, 35 cycle of denaturation stage for 20 s at 94 °C, annealing stage for 40 s at 52 °C, extension stage for 30 s at 72 °C, and the final elongation step was performed for 5 min at 72 °C. Following loading the PCR products on a 1% agarose gel with 0.5 µg/ml safe stain (Sinaclon, Iran), the gel was subjected to electrophoresis at 85 V for 60 min and the bands were then seen under UV light with the help of a gel documentation device. Positive and negative controls in this reaction include *K. pneumoniae* ATCC® BAA-1705™ and *K. pneumoniae* ATCC® BAA-1706™, respectively.

**Table 1:** Primers used in this study for the detection of the different carbapenemase genes

Genes	Size (bp)	Annealing (°C)	Nucleotide Sequences (5’-3’)
***Bla***_**VIM**_	390	54	F: GATGGTGTTTGGTCGCATA/R: CGAATGCGCAGCACCAG
***bla***_**NDM**_	621	57	F: GGTTTGGCGATCTGGTTTTC/R: CGGAATGGCTCATCACGATC
***bla***_**KPC**_	893	55	F: ATGTCACTGTATCGCCGTCT/R: TTTTCAGAGCCTTACTGCCC
***bla***_**OXA−48−like**_	744	58	F: TTGGTGGCATCGATTATCGG/R: GAGCACTTCTTTTGTGATGGC
***bla***_**IMP**_	740	49	F: TGAGCAAGTTATCTGTATTC/R: TTAGTTGCTTGGTTTTGATG

### Statistical analysis

The twentieth version of SPSS software (IBM Corporation, Armonk, NY, USA) was used for statistical analysis of the data. Results were presented as descriptive statistics in the form of relative frequencies. Values were expressed as percentages of the variables. An analysis of possible correlations between variables was conducted using Fisher’s exact test. The correlation was considered statistically significant if the *P*-value was less than 0.05.

## Results

### Distribution of the *K. pneumoniae* isolates in clinical samples

In our investigation, 100 *K. pneumoniae* isolates were collected from 57 urine samples, 27 blood samples, 13 wound samples, and 3 sputum samples. In total, 70.0% of the samples were collected from inpatients, while 30.0% were collected from outpatients.

### Antimicrobial susceptibility

**Fig. 1:**
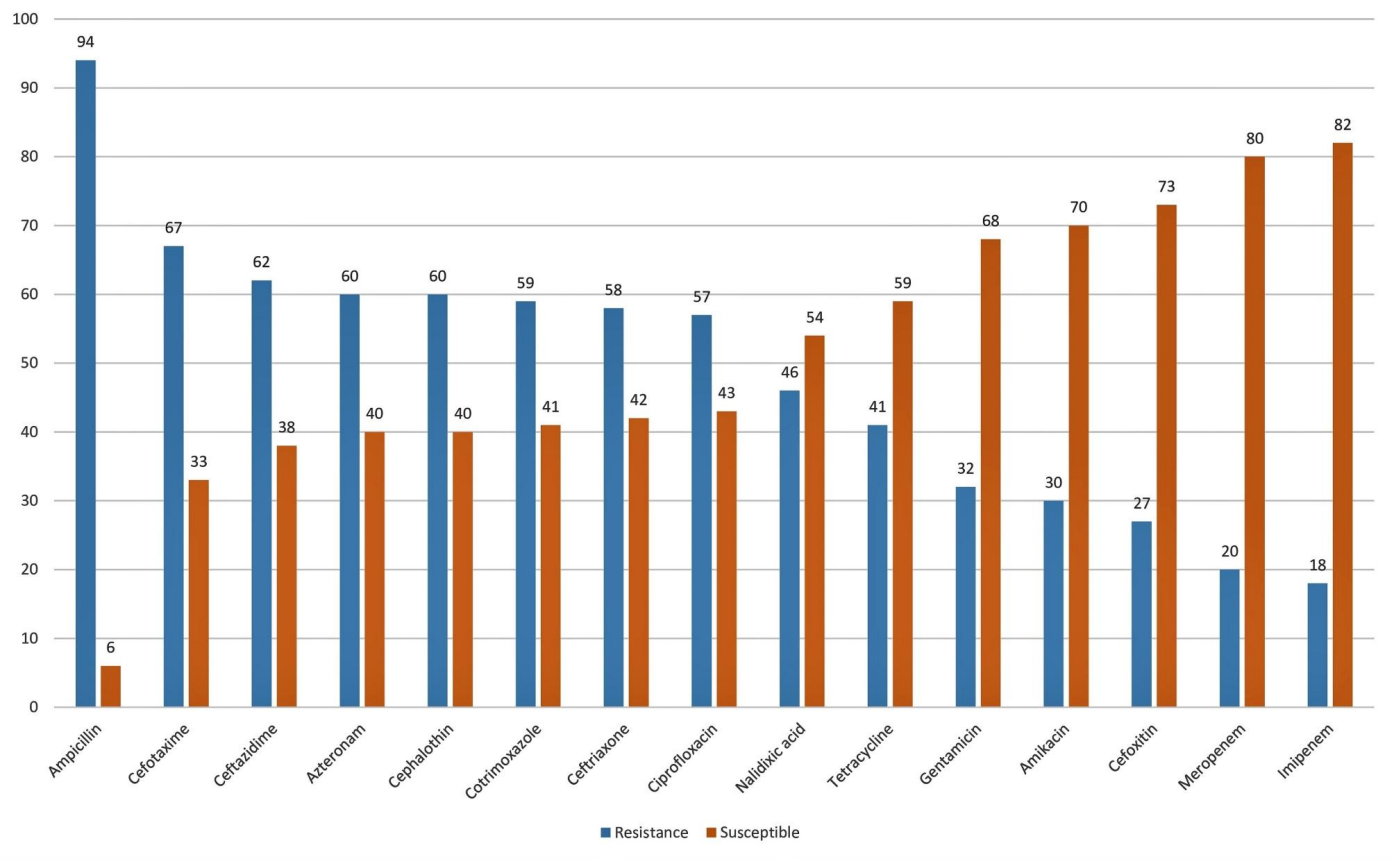
Antibiotic resistance percent rates in *Klebsiella pneumoniae* isolates collected from clinical samples in Tabriz, northwestern Iran

The results of AST of the 100 *K. pneumoniae* isolates against 15 antibiotics are shown in Fig. 1. Overall, the most resistance rates of *K. pneumoniae* isolates were related to ampicillin (94.0%), cefotaxime (67.0%) and ceftazidime (62.0%), and the lowest resistance rates were related to imipenem (18.0%) and meropenem (20.0%) antibiotics. In total, 25.0% of isolates were carbapenem-resistant, of which 13.0% were resistant to both imipenem and meropenem antibiotics. The carbapenem-resistant isolates were related to 18 (72.0%) males and 7 (28.0%) females. In total, 49.0% of *K. pneumoniae* isolates were MDR.

### Distribution of the carbapenemase genes

The distribution of different carbapenemase genes is presented in Table 2. In this study, among the 25 carbapenem-resistant isolates, the *bla*_VIM_, *bla*_KPC_, *bla*_OXA**−**48−like,_ and *bla*_IMP_ genes were detected by PCR. Eighteen isolates (72.0%) had the *bla*_KPC_ gene, 3 (12.0%) isolates had the *bla*_VIM_ gene, 1 (4.0%) isolate had the *bla*_IMP_ gene, and 1 (4.0%) isolate had the *bla*_OXA**−**48−like_ gene. Two carbapenem-resistant isolates did not carry any gene. The *bla*_NDM_ gene was not detected in any isolate. Coexistence of carbapenemase genes was also not detected in any isolate. The 18 *bla*_KPC_ positive *K. pneumoniae* were isolated from 10 urine, 5 blood, and 3 wound samples. Of 3 *bla*_VIM_ positive isolates, 2 were from urine and one from blood. The *bla*_IMP_ and *bla*_OXA_-_4− like_ positive isolates were from urine and blood, respectively.

**Table 2:** Distribution of *bla*_VIM_, *bla*_NDM_, *bla*_KPC_, *bla*_OXA_, and *bla*_IMP_ carbapenemase genes in the carbapenem-resistant *Klebsiella pneumoniae* isolates based on gender, referral type, and sample type

	Negative n (%)	*bla*_KPC_ n (%)	*bla*_VIM_ n (%)	*bla*_IMP_ n (%)	*bla*_OXA−48−like_ n (%)	*bla*_NDM_ n (%)	Total n (%)
Gender							
**Male**	34 (66.7)	13 (25.5)	2 (3.9)	1 (2.0)	1 (2.0)	0 (0.0)	51 (51.0)
**Female**	43 (87.8)	5 (10.2)	1 (2.0)	0 (0.0)	0 (0.0)	0 (0.0)	49 (49.0)
**Referral type (%)**,							
**Inpatient**	56 (80.0)	10 (14.3)	3 (4.3)	1 (1.4)	0 (0.0)	0 (0.0)	70 (70.0)
**Outpatient**	21 (70.0)	8 (26.7)	0 (0.0)	0 (0.0)	1 (3.3)	0 (0.0)	30 (30.0)
**Sample type (%)**,							
**Urine**	44 (77.2)	10 (17.5)	2 (3.5)	1 (1.8)	0 (0.0)	0 (0.0)	57 (57.0)
**Blood**	20 (74.1)	5 (18.5)	1 (3.7)	0 (0.0)	1 (3.7)	0 (0.0)	27 (27.0)
**Wound**	10 (76.9)	3 (23.1)	0 (0.0)	0 (0.0)	0 (0.0)	0 (0.0)	13 (13.0)
**Sputum**	3 (100.0)	0 (0.0)	0 (0.0)	0 (0.0)	0 (0.0)	0 (0.0)	3 (3.0)
**Total**	77 (77.0)	18 (18.0)	3 (3.0)	1 (1.0)	1 (1.0)	0 (0.0)	100 (100.0)

KPC: *Klebsiella pneumoniae* carbapenemase, OXA: oxacillinase, VIM: Verona integron-encoded metallo-β-lactamase, IMP: imipenamas, NDM: New Delhi metallo-β-lactamase

### Association of carbapenemase genes with antibiotic resistance patterns

The results of Fisher’s exact test showed that there was a significant association between the presence of carbapenemase genes with the resistance to meropenem (*P*-value = 0.000), imipenem (*P*-value = 0.000), cephalothin (*P*-value = 0.007), ceftazidime (*P*-value = 0.000), cefotaxime (*P*-value = 0.006), and ceftriaxone (*P*-value = 0.006).

## Discussion

In this study, the lowest resistance rates were found with the antibiotics imipenem (18.0%) and meropenem (20.0%). Also, 25.0% of the isolates were resistant to carbapenems. In contrast to our study, researchers found an unusual increase in carbapenem resistance in a retrospective cross-sectional study conducted from 2014 to 2018 in Saudi Arabia, with 38.4% for imipenem and 46.1% for meropenem [10]. However, the resistance rate to amikacin (36.3%) was lower, and the ciprofloxacin resistance was the same as in this study [10]. In another study conducted in Baluchistan in 2021, the lowest resistance was related to imipenem (0%), which was lower than in our study, and the highest resistance was related to cefotaxime (100.0%), which was higher than in our result [11]. In another study from Russia, the rate of carbapenem-resistance phenotypes (45.7%) was higher than in our study [12]. In contrast to this study, higher prevalence rates of CR-Kp isolates were found in the Iranian cities of Tehran (more than 60.0%) and Isfahan (more than 50.0%) [13, 14]. By comparing these studies, we concluded that the rate of CR-Kp in northwestern Iran was relatively lower than in other regions of the country. However, further studies with a larger sample size are needed to support these results. These findings seem to be useful for epidemiologists and physicians to improve their understanding of hospital-acquired infections and to implement antibiotic resistance control programs. In this study, *K. pneumoniae* isolates showed a high resistance rate (more than 50.0%) against third-generation cephalosporins that may be due to the presence of extended spectrum β-lactamase (ESBL) enzyme in the plasmids harbored by *K. pneumoniae* isolates [10, 11]. Also, this study revealed that aminoglycosides including amikacin and gentamicin were among the highly effective antibiotics against CR-Kp that was in line with a previous report from Saudi Arabia [10]. Also, Ferreira et al. [15] and Awoke et al. [16] reported the amikacin as one of the most effective antibiotics against K. pneumoniae isolates. Hence, aminoglycosides may be effective in the empirical treatment of infections caused by CR-Kp in northwestern Iran.

Another finding of this study was the frequency rate of 49.0% of MDR *K. pneumoniae*, which was lower than in previous studies from Brazil (84.0%) [15] and Ethiopia (98.5%) [16]. Compared with our results, Farhadi et al. [17] from Mazandaran province in northern Iran reported a higher prevalence (58.0%) of MDR *K. pneumoniae*. Antibiotic resistance patterns of *K. pneumoniae* isolates vary in different areas and studies due to differences in antibiotic prescribing, lack of drug resistance control programs, overuse of antibiotics in food and agricultural industries, and differences in epidemiology among regions. Data on *K. pneumoniae* and its antimicrobial resistance profiles in different geographic areas can help clinicians choose the best empiric antibiotic therapy.

In this study, the prevalence of *bla*_VIM_, *bla*_NDM_, *bla*_KPC_, *bla*_OXA_, and *bla*_IMP_ genes was investigated in CR-Kp isolates. The results showed that a total of 23.0% of *K. pneumoniae* isolates carried various carbapenemase genes. Also, *bla*_KPC_ (72.0%) was the most prevalent carbapenemase gene among CR-Kp isolates followed by *bla*_VIM_ (12.0%), *bla*_IMP_ (4.0%), and *bla*_OXA**−**48−like_ (4.0%) genes. However, the *bla*_NDM_ was not detected in any isolate. Coexistence of carbapenemase genes was also not detected in any isolate. In contrast to the current study, in a previous report from Busher in southern Iran, the rate of carbapenemase-producing *K. pneumoniae* (CP-Kp) was lower (7.9%), the *bla*_NDM−1_ (91.6%) and *bla*_OXA−48−like_ (33.3%) genes were the most prevalent carbapenemase genes among CP-Kp isolates, and the coexistence of *bla*_NDM_ and *bla*_OXA−48−like_ genes was present in 25.0% of the isolates [18]. In previous studies from Iran by Gheitani et al. [14] and Khorvash et al. [19], no CR-Kp isolate carried *bla*_KPC_, which was in contrast to the current study. However, similar to this study, Khorvash et al. [19] did not find *bla*_NDM_ gene in their isolates. Although the *bla*_NDM_ gene was not detected in this study, it has been speculated that Middle Eastern countries may serve as reservoirs for the spread of this type of carbapenemase [20]. Shahcheraghi et al. [21] have identified *bla*_NDM−1_ containing *K. pneumoniae* in Iran for the first time.

Pourgholi et al. [20] from Tehran, Iran (67.6%) and Ssekatawa et al. [8] from Uganda (36.4%) reported the *bla*_OXA−48−like_ as the most frequent carbapenemase in *K. pneumoniae* isolates, which was much higher than the current study (4.0%). There is ample evidence that the *bla*_OXA−48−like_ gene is present in several other countries, including Nepal [22] and South Africa [23]. However, this gene was not detected in the previous studies from Brazil [15] and China [24]. In this study, the *bla*_VIM_ and *bla*_IMP_ genes were detected in 3 (12.0%) and 1 (4.0%) isolates, respectively. In a previous study by Ragheb et al. [25] from Egypt, the *bla*_VIM_ gene was detected as the most frequent carbapenemase (84.62%) in *K. pneumoniae* isolates, which was higher than in this study. Likewise, they reported the *bla*_IMP_ gene in a higher proportion (58.97%) of isolates than in the current research [25]. In line with the current study, Bilal et al. [26] from Pakistan, reported low frequency of *bla*_IMP_ (7.2%) and *bla*_VIM_ (3.2%) genes in *K. pneumoniae* isolates. In contrast to this study, in a previous report from Turkey, the *bla*_KPC_ and *bla*_VIM_ genes were not detected in *Enterobacterales* isolates [27]. Likewise, no *bla*_IMP_, *bla*_VIM_, and *bla*_KPC_ genes were detected among clinical isolates of *K. pneumoniae* by Yasbolaghi Sharahi et al. [13] from Tehran, Iran that was in contrast to this study. In another study by Hosseinzadeh et al. [28] from Shiraz, southwestern Iran, *bla*_OXA−48−like_ and *bla*_NDM−1_ genes were found in 0.9% and 10.9% of *K. pneumoniae* isolates, respectively. These results confirmed the discrepancies in the distribution of various carbapenemase genes in different geographical areas. Reasons for discrepancies in the results include differences in the study population, the source of the isolates, and the technique used to identify the carbapenemases. In our study, no carbapenemase genes were found in 2 carbapenem-resistant isolates, suggesting that their resistance may be due to other mechanisms, such as production of AmpC beta-lactamases, ESBLs, efflux pumps, decreased permeability of outer membranes, absence of the CRISPR/Cas system, or possibly other carbapenemase genes that were not studied [20, 29, 30]. In this study, a total of 36.0% (n = 9/25) of CR-Kp isolates were detected in the outpatient department. In line with our results, a high rate of carbapenem-resistant *Enterobacterales* (57.5%) collected from the outpatient department has been previously reported by Abdelaziz [31] from Egypt. This can be a warning sign to implement surveillance programs to hinder any further spread of this strains. Presently, the only three nations that have an orderly organized national surveillance system are Thailand, the European Union, and the United States [32]. Gathering national information is necessary for establishing appropriate policy, revising lists of vital drugs for infection treatment, and assessing the effects of intervention strategies [33].

The WHO highlighted 12 antibiotic-resistant pathogens in 2017, including *K. pneumoniae*, to be the most threatening to human health [34]. Considering the increasing resistance of bacteria to many antibiotics, including carbapenems, more studies are necessary to achieve new treatment methods using plant metabolites or vaccine design [34, 35].

### Limitations

This study had several limitations, including the lack of sequencing of the carbapenemase gene and the failure to evaluate the clonal association of the CR-Kp isolates by a PCR-based method. Also, the other carbapenem resistance mechanisms, including efflux pumps expression and porin profiles of the isolates, were not investigated.

## Conclusion

This study showed that the prevalence of CP-Kp with carbapenemase genes was lower in northwestern Iran compared to other regions of the country. Moreover, *bla*_KPC_ was the most frequent carbapenemase gene. To prevent the further spread of CR-Kp in northwestern Iran, infection control measures, better screening methods, and optimal use of existing antibiotics should be emphasized.

## Data Availability

The data of the current study are available from the corresponding author on reasonable request.

## Abbreviations

AST: Antimicrobial susceptibility testing
CLSI: Clinical and Laboratory Standards Institute
MDR: Multidrug resistant
PCR: polymerase chain reaction
CR-Kp: Carbapenem-resistant *Klebsiella pneumoniae*

## References

[1] Choby J, Howard-Anderson J, Weiss D (2020). Hypervirulent *Klebsiella pneumoniae*–clinical and molecular perspectives. J Intern Med.

[2] Jafari-Sales A, Soleimani H, Moradi L (2020). Antibiotic resistance pattern in *Klebsiella pneumoniae* strains isolated from children with urinary tract Infections from Tabriz hospitals. Health Biotechnol Biopharma.

[3] Ramachandran G, Rajivgandhi GN, Murugan S, Alharbi NS, Kadaikunnan S, Khaled JM (2020). Anti-carbapenamase activity of *Camellia japonica* essential oil against isolated carbapenem resistant *Klebsiella pneumoniae* (MN396685). Saudi J Biol Sci.

[4] Hammoudi Halat D, Ayoub Moubareck C (2020). The current burden of carbapenemases: review of significant properties and dissemination among gram-negative bacteria. Antibiotics.

[5] Cowan ST. Cowan and Steel’s manual for the identification of medical bacteria. Cambridge university press; 2003.

[6] CLSI. Performance Standards for Antimicrobial Susceptibility Testing, 30th ed. CLSI supplement M100. Wayne, PA: Clinical and Laboratory Standards Institute; 2020.

[7] Alkofide H, Alhammad AM, Alruwaili A, Aldemerdash A, Almangour TA, Alsuwayegh A (2020). Multidrug-resistant and extensively drug-resistant *Enterobacteriaceae*: prevalence, treatments, and outcomes–a retrospective cohort study. Infect Drug Resist.

[8] Ssekatawa K, Byarugaba DK, Nakavuma JL, Kato CD, Ejobi F, Tweyongyere R (2021). Prevalence of pathogenic *Klebsiella pneumoniae* based on PCR capsular typing harbouring carbapenemases encoding genes in Uganda tertiary hospitals. Antimicrob Resist Infect Control.

[9] Shao C, Wang W, Liu S, Zhang Z, Jiang M, Zhang F (2021). Molecular epidemiology and drug resistant mechanism of carbapenem-resistant *Klebsiella pneumoniae* in elderly patients with Lower Respiratory Tract Infection. Front Public Health.

[10] Al-Zalabani A, AlThobyane OA, Alshehri AH, Alrehaili AO, Namankani MO, Aljafri OH (2020). Prevalence of *Klebsiella pneumoniae* antibiotic resistance in Medina, Saudi Arabia, 2014–2018. Cureus.

[11] Fatima S, Liaqat F, Akbar A, Sahfee M, Samad A, Anwar M (2021). Virulent and multidrug-resistant *Klebsiella pneumoniae* from clinical samples in Balochistan. Int Wound J.

[12] Fursova AD, Fursov MV, Astashkin EI, Novikova TS, Fedyukina GN, Kislichkina AA (2022). Early response of antimicrobial resistance and virulence genes expression in classical, hypervirulent, and hybrid hvKp-MDR *Klebsiella pneumoniae* on antimicrobial stress. Antibiotics.

[13] Yasbolaghi Sharahi J, Hashemi A, Ardebili A, Davoudabadi S (2021). Molecular characteristics of antibiotic-resistant *Escherichia coli* and *Klebsiella pneumoniae* strains isolated from hospitalized patients in Tehran, Iran. Ann Clin Microbiol Antimicrob.

[14] Gheitani L, Fazeli H, Moghim S, Isfahani BN (2018). Frequency determination of carbapenem-resistant *Klebsiella pneumoniae* (CRKP) isolated from hospitals in Isfahan of Iran and evaluation of synergistic effect of colistin and meropenem on them. Cell Mol Biol.

[15] Ferreira RL, da Silva B, Rezende GS, Nakamura-Silva R, Pitondo-Silva A, Campanini EB (2019). High prevalence of multidrug-resistant *Klebsiella pneumoniae* harboring several virulence and β-lactamase encoding genes in a Brazilian intensive care unit. Front Microbiol.

[16] Awoke T, Teka B, Seman A, Sebre S, Yeshitela B, Aseffa A (2021). High prevalence of multidrug-resistant *Klebsiella pneumoniae* in a tertiary care hospital in Ethiopia. Antibiotics.

[17] Farhadi M, Ahanjan M, Goli HR, Haghshenas MR, Gholami M (2021). High frequency of multidrug-resistant (MDR) *Klebsiella pneumoniae* harboring several β-lactamase and integron genes collected from several hospitals in the north of Iran. Ann Clin Microbiol Antimicrob.

[18] Latifi B, Tajbakhsh S, Askari A, Yousefi F (2020). Phenotypic and genotypic characterization of carbapenemase-producing *Klebsiella pneumoniae* clinical isolates in Bushehr province, Iran. Gene Rep.

[19] Khorvash F, Yazdani MR, Soudi AA, Shabani S, Tavahen N (2017). Prevalence of acquired carbapenemase genes in *Klebsiella Pneumoniae* by multiplex PCR in Isfahan. Adv Biomed Res.

[20] Pourgholi L, Farhadinia H, Hosseindokht M, Ziaee S, Nosrati R, Nosrati M (2022). Analysis of carbapenemases genes of carbapenem-resistant *Klebsiella pneumoniae* isolated from Tehran heart center. Iran J Microbiol.

[21] Shahcheraghi F, Nobari S, Rahmati Ghezelgeh F, Nasiri S, Owlia P, Nikbin VS (2013). First report of New Delhi metallo-beta-lactamase-1-producing *Klebsiella pneumoniae* in Iran. Microb Drug Resist.

[22] Gurung S, Kafle S, Dhungel B, Adhikari N, Shrestha UT, Adhikari B (2020). Detection of OXA-48 gene in carbapenem-resistant Escherichia coli and Klebsiella pneumoniae from urine samples. Infect Drug Resist.

[23] Perovic O, Ismail H, Quan V, Bamford C, Nana T, Chibabhai V (2020). Carbapenem-resistant *Enterobacteriaceae* in patients with bacteraemia at tertiary hospitals in South Africa, 2015 to 2018. Eur J Clin Microbiol Infect Dis.

[24] Li J, Li C, Cai X, Shi J, Feng L, Tang K (2019). Performance of modified carbapenem inactivation method and inhibitor-based combined disk test in the detection and distinguishing of carbapenemase producing *Enterobacteriaceae*. Ann Transl Med.

[25] Ragheb SM, Tawfick MM, El-Kholy AA, Abdulall AK (2020). Phenotypic and genotypic features of *Klebsiella pneumoniae* harboring carbapenemases in Egypt: OXA-48-like carbapenemases as an investigated model. Antibiotics.

[26] Bilal H, Zhang G, Rehman T, Han J, Khan S, Shafiq M (2021). First report of *bla*_NDM–1_ bearing IncX3 plasmid in clinically isolated ST11 *Klebsiella pneumoniae* from Pakistan. Microorganisms.

[27] Cayci YT, Biyik I, Korkmaz F, Birinci A (2021). Investigation of NDM, VIM, KPC and OXA-48 genes, blue-carba and CIM in carbapenem resistant *Enterobacterales* isolates. J Infect Dev Ctries.

[28] Hosseinzadeh Z, Ebrahim-Saraie HS, Sarvari J, Mardaneh J, Dehghani B, Rokni-Hosseini SM (2018). Emerge of *bla*_NDM–1_ and *bla*_OXA–48–like_ harboring carbapenem-resistant *Klebsiella pneumoniae* isolates from hospitalized patients in southwestern Iran. J Chin Med Assoc.

[29] Al-Ouqaili MT, Al-Taei SA, Al-Najjar A (2018). Molecular detection of medically important carbapenemases genes expressed by metallo-β-lactamase producer isolates of *Pseudomonas aeruginosa* and *Klebsiella pneumoniae*. Asian J Pharm.

[30] Jwair NA, Al-Ouqaili MT, Al-Marzooq F (2023). Inverse association between the existence of CRISPR/Cas systems with antibiotic resistance, extended spectrum β-lactamase and carbapenemase production in multidrug, extensive drug and pandrug-resistant *Klebsiella pneumoniae*. Antibiotics.

[31] Abdelaziz NA (2022). Phenotype-genotype correlations among carbapenem-resistant *Enterobacterales* recovered from four Egyptian hospitals with the report of SPM carbapenemase. Antimicrob Resist Infect Control.

[32] Soltani J, Poorabbas B, Miri N, Mardaneh J (2016). Health care associated Infections, antibiotic resistance and clinical outcome: a surveillance study from Sanandaj, Iran. World J Clin Cases.

[33] Poorabbas B, Mardaneh J, Rezaei Z, Kalani M, Pouladfar G, Alami MH (2015). Nosocomial Infections: Multicenter surveillance of antimicrobial resistance profile of *Staphylococcus aureus* and Gram negative rods isolated from blood and other sterile body fluids in Iran. Iran J Microbiol.

[34] Dey J, Mahapatra SR, Raj TK, Kaur T, Jain P, Tiwari A, Patro S, Misra N, Suar M (2022). Designing a novel multi-epitope vaccine to evoke a robust immune response against pathogenic multidrug-resistant *Enterococcus faecium* bacterium. Gut Pathog.

[35] Mahapatra SR, Dey J, Raj TK, Kumar V, Ghosh M, Verma KK (2022). The potential of plant-derived secondary metabolites as novel drug candidates against *Klebsiella pneumoniae*: molecular docking and simulation investigation. S Afr J Bot.

